# The colonial cnidarian *Hydractinia*

**DOI:** 10.1186/s13227-020-00151-0

**Published:** 2020-03-26

**Authors:** Uri Frank, Matthew L. Nicotra, Christine E. Schnitzler

**Affiliations:** 1 Centre for Chromosome Biology, School of Natural Sciences, National University of Ireland Galway, Galway, Ireland; 2 Departments of Surgery and Immunology, Center for Evolutionary Biology and Medicine, Thomas E. Starzl Transplantation Institute, University of Pittsburgh, Pittsburgh, PA 15261 USA; 3 Whitney Laboratory for Marine Bioscience, University of Florida, St. Augustine, FL 320803 USA; 4 Department of Biology, University of Florida, Gainesville, FL 32611 USA

**Keywords:** Cnidaria, Hydrozoa, Allorecognition, Regeneration, Stem cells, CRISPR

## Abstract

*Hydractinia*, a genus of colonial marine cnidarians, has been used as a model organism for developmental biology and comparative immunology for over a century. It was this animal where stem cells and germ cells were first studied. However, protocols for efficient genetic engineering have only recently been established by a small but interactive community of researchers. The animal grows well in the lab, spawns daily, and its relatively short life cycle allows genetic studies. The availability of genomic tools and resources opens further opportunities for research using this animal. Its accessibility to experimental manipulation, growth- and cellular-plasticity, regenerative ability, and resistance to aging and cancer place *Hydractinia* as an emerging model for research in many biological and environmental disciplines.

## Natural habitat and lifecycle

**Fig. 1:**
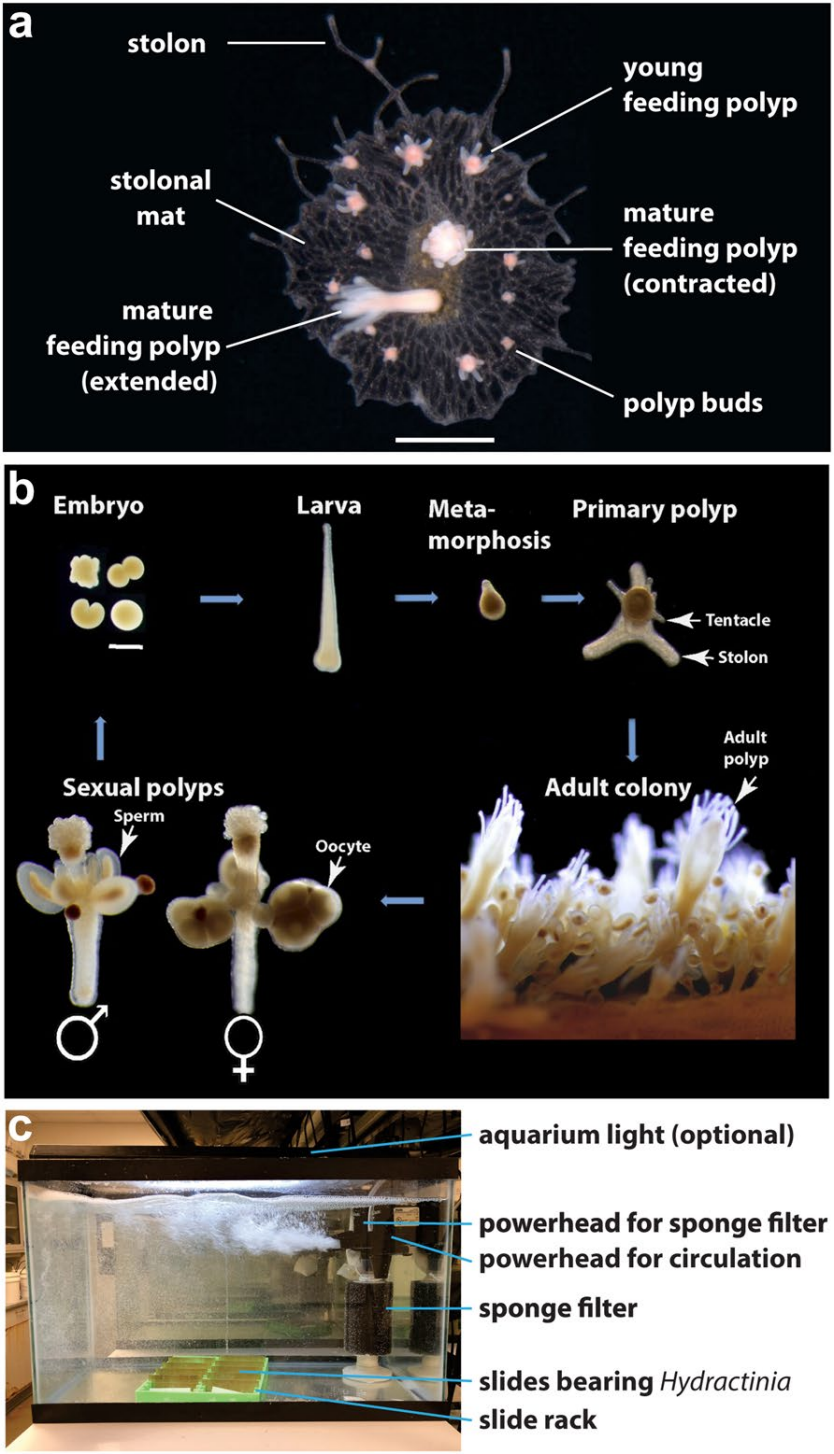
*Hydractinia* morphology, life history, and culture. **a** Colony growing on a microscope slide. Major morphological structures are labeled. This colony was explanted from a larger colony. The yellow-brown rectangle at the center is a layer of chitin that is slowly deposited below the mat as the colony grows and indicates the outline of the original explant. Scale bar = 1 mm. **b** Life cycle of *Hydractinia*. **c** Typical setup of a 39-L glass aquarium for culturing *Hydractinia* (image in **b** from Ref. [18] and licensed under CC BY 4.0 (link: https://creativecommons.org/licenses/by/4.0/))

*Hydractinia symbiolongicarpus* and *H. echinata* are sister species of colonial hydrozoan cnidarians. *H. symbiolongicarpus* occurs along the eastern coast of North America, from Maine to South Carolina [1]. *H. echinata* is found along North European Atlantic coasts [2]. In the field, they are found exclusively on gastropod shells occupied by hermit crabs (e.g., *Pagurus longicarpus*). Colonies consist of polyps specialized for feeding, reproduction, or defense, which grow from a sheet of tissue called the stolonal mat (Fig. 1a). Unlike many of its hydrozoan relatives, *Hydractinia* does not produce a free-living medusa stage (jellyfish). Instead, gametes mature in a rudimentary medusoid that remains attached to sexual polyps (Fig. 1b). All polyps within a colony are clonally derived and therefore genetically identical. The mat consists of two epidermal cell layers, which sandwich a network of gastrodermal canals connecting polyps to each other and forming a gastrovascular system. Colonies grow by expanding the edge of the mat or by elongating individual stolons, extensions of gastrovascular canals encased in a thin, chitinous integument called the periderm. Colonies are dioecious and spawn about 90 min after first light. Eggs sink to the bottom after fertilization and develop into a planula larva within 2–3 days (Fig. 1b). Mature larvae latch onto a passing hermit crab shell by firing nematocysts located in their posterior ends [3]. Once on the shell, the larvae metamorphose into a primary polyp in response to a bacterial cue [4]. The juvenile colony then grows as described above, frequently covering the entire shell.

## Lab culture and field collection

*Hydractinia* can be cultured in the lab with supplies available at most aquarium stores (Fig. 1c). A typical setup is a 39-L glass aquarium filled with any commercial artificial seawater (29–32 ppt) and maintained at 18–22 °C. Colonies grow best with ample water movement, thus a power head (usually one designed for a 110 L tank) is attached to the side of the tank. *Hydractinia* is sensitive to the accumulation of ammonia and nitrites. Biological filtration is therefore provided with an external filter or an internal sponge filter and second power head. Phosphates can also inhibit colony growth but are controlled by placing small bags of phosphate absorbing media in each tank. With this in place, a weekly 25% water change is enough to maintain healthy colonies.

To establish a *Hydractinia* culture from a field-collected animal, a piece of the colony bearing several feeding polyps is excised from its shell with a sharp blade and then tied with thread to a standard 25 × 75 mm glass microscope slide. The thread is removed after the explant attaches to the slide. This colony can then be propagated indefinitely by explanting it onto new slides. The slides can be stored in a histology slide box with the cover removed and the bottom cut out, which is placed at the bottom of the tank.

*Hydractinia* can also be bred in the lab. This is done by keeping them on a regular dark:light cycle (e.g., 8 h:16 h). Approximately an hour and a half after turning on the lights, eggs and sperm are spawned and can be combined in artificial seawater in a Petri dish, where fertilized eggs begin embryonic development. After 3–4 days, the resulting larvae may be induced to settle by incubating them for 2–3 h in 100 mM CsCl and subsequently placing them on a new microscope slide. There, larvae metamorphose into a primary polyp, which is competent to feed within 1–3 days post-metamorphosis. Despite the predictable, light-induced spawning, spawning may occasionally occur at other times. It is therefore advisable to keep male and female colonies in separate tanks to prevent the uncontrolled production of larvae.

Laboratory cultures of *Hydractinia* fare well on a diet of 4-day-old *Artemia* nauplii, which they receive three times per week. When many embryos are required for an experiment, we have found it beneficial to supplement this diet with pureed oysters twice a week. Colonies receiving this diet release gametes more reliably, in greater quantities, and with higher quality.

Today, most laboratories studying *Hydractinia symbiolongicarpus* work with strains derived from a single population in New Haven Harbor, Connecticut. The primary strain is a male colony, called 291-10, which is particularly vigorous in laboratory culture and, for this reason, was the animal chosen for the *Hydractinia* genome project (see below). Several female strains (e.g., 295-8) are also in use and their genome sequencing is in progress. Transgenic/mutant strains, derived from crossing 291-10 to a female strain, have also been established. All strains are available by request from our labs. Some European researchers use *H. echinata*, for which a full genome sequence has been generated as well; however, no selected laboratory strains exist for this species and its maintenance is more challenging.

## Major interests and research questions

**Fig. 2:**
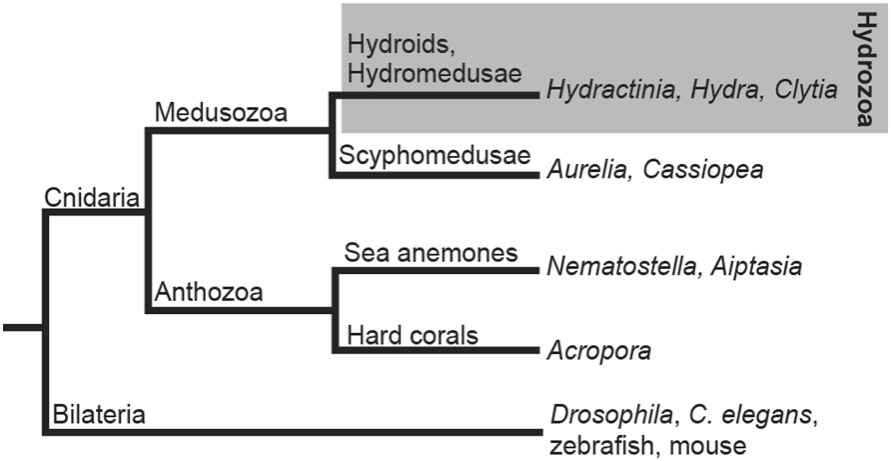
Cladogram showing evolutionary relationships between *Hydractinia* and other model organisms

Cnidarians are an interesting and highly diverse group of animals. This phylum diverged from the lineage leading to bilaterian animals (that includes flies, worms, and vertebrates) at least 600 million years ago [5], providing sufficient time for substantial diversification within the cnidarian lineage (Fig. 2). Most extant cnidarians share a body wall consisting of an epithelial bilayer, a gastric cavity, and a unique cell type—the stinging cell or cnidocyte (also known as nematocyte) from which the phylum name derives. Cnidarians are phylogenetically positioned as the sister group to bilaterians [6]; therefore, studying biological phenomena in cnidarians can provide insight into their origin and how they have changed over evolutionary time between and within phyla. The past two decades have brought substantial progress in cnidarian molecular biology and genetics, enabling functional genetic studies at least in some cnidarian representatives [7]. Overall, cnidarians’ relative morphological simplicity, sequenced genomes [8–10], amenability to genetic manipulation [11–13], and phylogenetic position promise a fruitful future in research on these animals that will inform areas spanning all the way from evolutionary biology to biomedical sciences.

Current research on *Hydractinia* focuses on a number of topics, including embryonic development [14], neurogenesis [15, 16], stem cells, germ cells, and regeneration [17–20], allorecognition [21], metabolism [22], immunity [23], and natural product chemistry [24]. Allorecognition refers to the ability to discriminate ‘self’ from ‘non-self’ within the same species, a phenomenon observed in most colonial cnidarians, but not in *Hydra* or *Nematostella*, the two most commonly used cnidarian model systems for molecular work. At present, *Hydractinia* is the only cnidarian in which genes controlling allorecognition have been identified and functionally characterized [25].

Other areas of interest are stem cells and regeneration. These topics have been well studied in *Hydra* [26, 27] and are emerging topics for *Nematostella* researchers too [28, 29]. Interestingly, data published to date suggest that both stem cell behavior and the mode of regeneration differ substantially between cnidarian species [18, 28, 30]. For example, hydrozoan neuronal cells derive from migratory i-cells, whereas in anthozoans, neural progenitor cells are epithelial [16]. As to regeneration modes, *Hydra* can reform the main head structures following decapitation in the absence of cell proliferation whereas in *Hydractinia* and *Nematostella* cell proliferation is essential for regeneration [18]. These findings highlight the importance of studying more than one animal in order to prevent false conceptual generalizations and underestimation of the complexity underlying biological phenomena.

*Hydractinia* does not show any evidence for age-related deterioration [31], is highly resistant to ionizing irradiation [18], and develops tumors only very rarely following genetic manipulation [19] but not spontaneously. These features are consistent with high genomic stability in this animal, a feature that remains to be investigated.

## Experimental approaches

Manipulating gene expression has so far only been established in four cnidarians: *Hydra*, *Nematostella*, *Hydractinia*, and *Clytia* [11–13, 32]. This can be done either by permanent modification of the animal’s genome or by transient interference with specific gene products. Both approaches have their pros and cons and their usage depends on the type of experiment being conducted and availability of appropriate protocols for a given species and life stage.

**Fig. 3:**
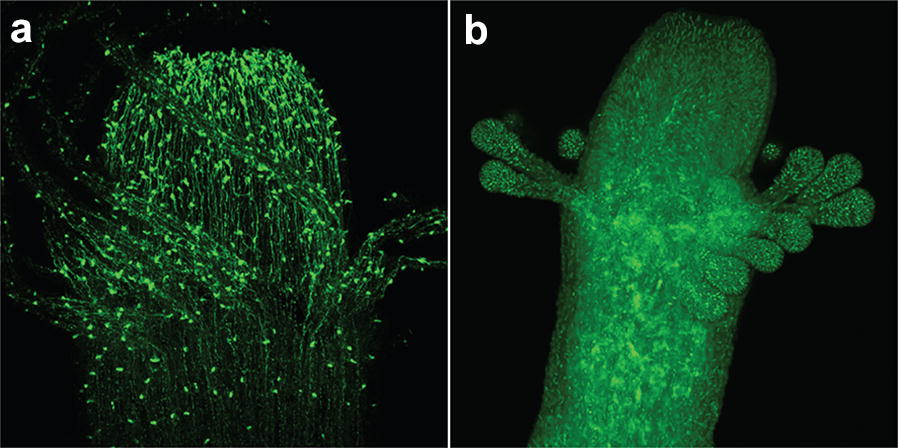
Live imaging of transgenic *Hydractinia* gastrozooids. **a** A polyp expressing eGFP under an RFamide precursor promoter, labeling a subset of neurons. The animal was created via random integration of a circular DNA plasmid. **b** A polyp expressing eGFP under the endogenous *Eef1a* promoter. The animal was created using CRISPR/Cas9 to target integration of the eGFP coding sequence into the *Hydractinia Eef1a* locus (image from Ref. [34] and licensed under CC BY 4.0 (link: https://creativecommons.org/licenses/by/4.0/))

The most common approach in *Hydractinia* is microinjection of nucleic acids and/or proteins into the zygote. *Hydractinia* spawning is light-induced without the need for any further induction [33]. Eggs are not embedded in jelly and can be directly microinjected upon fertilization [12]. Electroporation techniques are currently being developed in the authors’ labs with promising results. Circular plasmids readily integrate into the *Hydractinia* genome [12]. The site of integration is unknown, but the process is highly efficient; in excess of 80% of injected embryos become transgenic in the hands of experienced researchers. This approach has been used to create fluorescent reporter lines for many developmental genes and cell type-specific markers (e.g., Fig. 3). A more targeted way to genetically manipulate the animals is provided by CRISPR–Cas9 technology. In *Hydractinia*, this is performed by microinjecting site-specific short guide RNAs (sgRNA) together with recombinant Cas9 to generate loss-of-function mutations [16, 20]. Adding to the injecting cocktail a plasmid including a fragment of DNA, flanked by two homology arms, can be used for targeted knock-in of fragments [34]. As with all plasmids, this DNA could also integrate randomly into the genome. Designing the injected DNA such that it must rely on the promotor of the target gene limits the likelihood that it would be expressed if integrating non-specifically.

Gene expression manipulation without genetic alteration can be achieved by injecting short hairpin RNA (shRNA) [20] or morpholino oligonucleotides [35] to lower expression of genes, or synthetic RNA to overexpress them (Török et al. unpublished data). Finally, incubating polyps in seawater containing double stranded RNA (dsRNA) transiently lowers the expression of the corresponding gene, albeit with low efficiency [36].

*Hydractinia* is also unique among model cnidarians for being the only species in which a forward genetic approach has been used to identify the genetic basis of a phenotype. The reasons for this are almost entirely logistical. First, *Hydractinia* colonies can produce hundreds of embryos per day, making it possible to quickly generate large populations of bred animals. Second, the animals grow as encrustations on a surface that can be labeled, making it possible to co-culture large populations of genetically distinct animals in a small number of tanks. To date, forward genetic approaches have been used to identify genes responsible for allorecognition [37–39] and sex determination (Nicotra, unpublished data). Given the availability of a sequenced genome and the cost efficiency of high-throughput genotyping, it seems feasible to consider mutagenesis screens as well.

An additional experimental approach in *Hydractinia* is grafting of tissues. This can be done for, e.g., introducing transgenic cells into a colony [20]. Grafting of tissues from genetically distinct individuals requires at least partial matching of allorecognition alleles to prevent allogeneic rejection [25].

Single-cell RNA sequencing methods are also under development in our labs with the first single-cell sequencing libraries giving encouraging results. Our current goal is to develop a robust cellular atlas to define major cell types and subtypes in *Hydractinia* and to identify marker genes for all cell types as was recently done in *Hydra* and *Nematostella* [40, 41]. With a robust genome and cellular atlas in place, *Hydractinia* will be poised to answer biological questions in a more comprehensive way. Flow cytometry and fluorescence activated cell sorting (FACS) protocols are available [20], and together with many transgenic reporter strains it allows for generating cell type-specific transcriptomes following FACS-sorting of defined cell populations.

As with any model organism, *Hydractinia* has limitations. Perhaps most obvious one is that it lacks a medusa stage, so researchers interested in this feature must look elsewhere, notably to the hydroid *Clytia* and the scyphozoan *Aurelia*. The existing *Hydractinia* research community also remains small compared to that for *Hydra* and *Nematostella*, so the availability of shared reagents and techniques is somewhat more limited. This concern is increasingly mitigated by additional labs beginning to study *Hydractinia*, and an upsurge in crosstalk between researchers.

## Research community and resources

The *Hydractinia* research community is relatively small but growing as *Hydractinia* is gaining recognition as a tractable cnidarian research model. A recent NSF Enabling Discovery through GEnomic Tools (EDGE) grant has been awarded to the authors, ensuring that the genetic toolkit and community of *Hydractinia* researchers will continue to blossom and grow. Current resources include high-quality genomes and transcriptomes from both *Hydractinia symbiolongicarpus* and *H. echinata*. Draft Illumina genome and transcriptome assemblies are publicly available through the *Hydractinia* Genome Project Portal (https://research.nhgri.nih.gov/hydractinia/), and long-read PacBio genome assemblies for both species are forthcoming (Schnitzler et al. unpublished data). With an estimated genome size of 774 Mb for *H. echinata* and 514 Mb for *H. symbiolongicarpus*, the *Hydractinia* genomes are larger than the genome of *Nematostella* (329 Mb) but smaller than that of *Hydra* (1086 Mb). Annotated reference genomes and transcriptomes can be used for mapping standard RNA sequencing data [20]. Laboratory selected, fast-growing strains are available to anyone. We are developing a community portal at www.hydractinia.org to be completed in the coming months, which will link to written and video-based protocols and to a community forum, and provide an online form to request animals. Newcomers to the field are encouraged to attend the two biennial research conferences, the American Cnidofest [42] and the European Tutzing meeting [43].

## Data Availability

Not applicable.
